# Temporal Dynamics and Uptake Mechanisms of Carbonated Hydroxyapatite Nanoparticles in Murine F‑OST Cells

**DOI:** 10.1021/acsomega.5c12494

**Published:** 2026-03-18

**Authors:** Julliana Helena de Souza Sobrinho, Marcel Guimarães Martins, Grasiella Ventura Matioszek, Alexandre Malta Rossi, Bruno de Almeida Carlos de Carvalho Pontes, Fabrício Frizera Borghi, Danielle Cabral Bonfim, Sara Gemini-Piperni

**Affiliations:** † 125296 University of Grande Rio, Duque de Caxias, Rio de Janeiro 25075-142, Brazil; ‡ MAGTECH BRASIL, Business Incubator of Federal Fluminense University, Rio de Janeiro 24220-000, Brazil; § Institute of Biomedical Sciences − ICB, 28125 Federal University of Rio de Janeiro, Rio de Janeiro 21941-590, Brazil; ∥ 74350 Brazilian Center of Physical Research − CBPF, Botafogo, Rio de Janeiro, Rio de Janeiro 22290-180, Brazil; ⊥ Institute of Biomedical Sciences − ICB and National Center for Structural Biology and Bioimaging − CENABIO, Federal University of Rio de Janeiro, Rio de Janeiro 21941-590, Brazil; # Institute of Physics − ICB, Federal University of Rio de Janeiro, Rio de Janeiro 21941-590, Brazil

## Abstract

Carbonated hydroxyapatite (CarboHA) is a biomaterial gaining attention for its biocompatibility, bioactivity, and expanding applications in bone regeneration, aesthetic biostimulation, dentistry, and drug delivery systems. However, the impact of synthesis conditions on its physicochemical properties and cellular internalization mechanisms remains poorly understood. In this study, CarboHA nanoparticles were synthesized using a wet-chemistry method at three temperatures: 5 °C, 37 °C, and 90 °C. The particles were characterized by X-ray diffraction (XRD), Fourier transform infrared spectroscopy (FTIR), transmission electron microscopy (TEM), and zeta potential analysis. To enable fluorescent tracking during internalization studies in primary murine osteoblast (F-OST) cells, rhodamine was adsorbed onto the nanoparticles. Endocytic pathways were examined using selective inhibitors for clathrin-mediated, caveolin-mediated, lipid raft-mediated endocytosis, macropinocytosis, and phagocytosis and quantified via fluorescence microscopy and image analysis. The synthesis temperature significantly influenced crystallinity and morphology, which in turn dictated cell uptake mechanisms. Single-particle optical tweezers assays revealed increased adhesion times with higher synthesis temperatures. CarboHA synthesized at 5 °C produced smaller, less crystalline particles internalized predominantly through clathrin- and caveolin-mediated pathways, whereas highly crystalline nanoparticles produced at 90 °C favored macropinocytosis and phagocytosis. CarboHA synthesized at 37 °C demonstrated intermediate behavior, engaging multiple internalization routes. Together, these findings establish a clear relationship between synthesis temperature, nanostructural features, and cellular adhesion/internalization mechanisms, highlighting how controlling synthesis conditions enables the design of CarboHA-based materials optimized for specific biomedical applications, including resorbable bone grafts with tunable remodeling profiles and nanocarriers engineered for targeted intracellular delivery.

## Graphical abstract

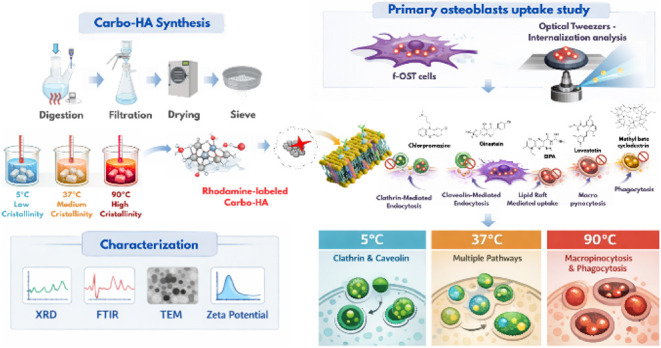

## 1 Introduction

Bone tissue loss due to trauma, degenerative diseases, congenital defects, or aging presents a growing clinical challenge worldwide, worsened by increasing life expectancy and the rising number of elderly individuals.^1,2^ As a load-bearing tissue with limited regenerative capacity in large defects, bone often requires specialized interventions to restore its structure and function. Currently, autologous bone grafting is regarded as the gold standard for bone repair due to its histocompatibility, and its inherent osteogenic, osteoinductive, and osteoconductive properties.^3^

However, this method has significant drawbacks, including donor site morbidity, infection risk, prolonged recovery time, pain, inflammation, and increased surgical costs.^4,5^

In response to these limitations, synthetic biomaterials have emerged as promising alternatives for bone regeneration. Among these, calcium phosphate-based ceramics, particularly hydroxyapatite (HA), have attracted significant attention due to their compositional similarity to the mineral phase of bone, as well as their excellent biocompatibility, bioactivity, and osteoconductivity.^6^ Hydroxyapatite’s utility in regenerative medicine extends beyond simple compositional mimicry; nanostructured HA particles can engage in complex interactions with cells, including membrane binding, uptake, and intracellular processing. Recent work has shown that HA nanoparticles not only bind to cell membranes and become internalized into vesicular compartments, but also undergo intracellular degradation, which may influence calcium signaling and subsequent cellular responses, illustrating the intricate relationship between material properties and biological fate.^7^

The morphologic characteristics of HA particles, such as size and shape, have been shown to alter the balance of cellular uptake routes and subsequent intracellular localization, highlighting the importance of tailoring nanoparticle design to elicit specific cellular interactions.^8^ Furthermore, HA’s ability to incorporate various ionic substitutions allows for fine-tuning of its solubility, mechanical properties, and biological performance.^9,10^ Ionic substitution (e.g., carbonate, strontium, magnesium) can adjust HA’s reactivity and degradation behavior to more closely mimic native bone mineral, enhancing its bioactivity and potential for controlled integration and remodeling. Recent advances have demonstrated that such compositional modulations, when combined with nanoscale structuring, can improve cell adhesion, osteogenic signaling, and overall regenerative outcomes.^11^

One noteworthy modification is carbonate substitution, which increases HA’s solubility and enhances its similarity to biological apatite, the naturally carbonated form. This characteristic improves the material’s resorption profile and facilitates ion exchange, which are vital for stimulating osteogenesis and enhancing clinical outcomes.^10^ Consequently, carbonated hydroxyapatite (CarboHA) has been extensively researched for applications in bone tissue engineering.

The synthesis of nanostructured CarboHA is crucial for determining its biological performance. High-temperature methods yield highly crystalline materials with excellent mechanical stability but often result in low solubility and poor biodegradability, which can restrict their bioactivity.^12^ In contrast, low-temperature wet synthesis methods—conducted under conditions closer to physiological temperature—produce CarboHA with a higher surface area, lower crystallinity, and improved solubility. These characteristics are particularly important for enhancing biological interactions, promoting cellular adhesion and proliferation, and ultimately facilitating bone regeneration.^13^

Despite the broad application of CarboHA -based materials in regenerative medicine, the precise mechanisms by which CarboHA nanoparticles interact with cells remain poorly understood, especially concerning their internalization pathways and intracellular trafficking. This knowledge gap is significant because the route of cellular uptake affects not only the bioavailability and distribution of the nanoparticles but also essential cellular processes, including differentiation, matrix mineralization, and immune response.^14^

Understanding these nano-bio interactions is crucial—not only for optimizing the design of CarboHA -based biomaterials but also for addressing regulatory challenges associated with nanotechnology in healthcare. Regulatory agencies, including Brazilian ANVISA and US FDA, increasingly demand comprehensive data on the physicochemical characterization, cellular interactions, and potential toxicological impacts of nanomaterials, recognizing that nanoscale properties can induce behaviors distinct from their micro- or macroscale counterparts.^15^ Moreover, the market is projected to experience substantial growth in nanostructured synthetic biomaterials due to their enhanced performance, improved patient acceptance, and reduced risks compared to traditional grafting methods

Therefore, gaining a deeper understanding of how synthesis parameters, especially temperature, affect the physicochemical properties of carbonated hydroxyapatite (CarboHA) and its cellular interaction is essential for optimizing biomaterial performance and advancing regulatory frameworks for nanostructured materials. The present study aims to evaluate the adhesion, internalization, and intracellular trafficking of CarboHA nanoparticles synthesized via the wet route at 5 °C, 37 °C, and 90 °C in primary murine osteoblast cultures (F-OST). We also characterize their physicochemical properties after exposure to culture medium and investigate the endocytic pathways involved in their uptake.

## 2 Experimental Section

### 2.1 Synthesis of Carbo-Hydroxyapatite Nanoparticles

Carbonated hydroxyapatite (CarboHA) was synthesized using a wet precipitation method adapted from a previously published procedure developed by collaborators and later reported by Anjos et al. (2019). The synthesis was carried out using the following reagents: calcium nitrate tetrahydrate (Ca­(NO_3_)_2_·4H_2_O) at a concentration of 0.21 mol/L, ammonium hydrogen phosphate ((NH_4_)_2_HPO_4_) at 0.09 mol/L, and ammonium carbonate ((NH_4_)_2_CO_3_) at 0.033 mol/L, maintaining a pH of 12. The reactions were carried out at three different temperatures: 5 °C (for 3 h), 37 °C (for 2 h), and 90 °C (for 2 h). The resulting precipitates were washed, vacuum-filtered, and dried using lyophilization at 5 °C and oven drying at 50 °C for 12 h for the samples synthesized at 37 and 90 °C. The powders were then sieved to a size of 75 μm. All syntheses were conducted in triplicate to ensure reproducibility.

### 2.2 Fluorescent Labeling

To enable tracking of internalization, CarboHA was fluorescently labeled with Rhodamine. A stock solution of 0.5 mg/mL Rhodamine was prepared, and 200 mg of CarboHA was incubated with 1 mL of this solution for 2 h while stirring. The samples were washed until the supernatant was clear and then dried. The labeling efficiency was confirmed through fluorescence microscopy, Fourier-transform infrared spectroscopy (FTIR), and zeta potential analysis.

### 2.3 Nanoparticle Exposure to Culture Medium

Nanoparticles at a concentration of 1 mg/mL were incubated in either water or DMEM supplemented with 10% fetal bovine serum (FBS) and 1% penicillin-streptomycin (PS) for 24 h at 37 °C under agitation. Following this, the samples were subjected to centrifugation at 300 RCF for 5 min and washed three times with Milli-Q water before being dried for 12 h.

### 2.4 Physicochemical Characterization

The physicochemical characterization of the carbonated hydroxyapatite (CarboHA) nanoparticles was conducted both before and after exposure to the cell culture medium to evaluate possible structural or surface modifications induced by biological conditions. A combination of complementary analytical techniques was employed to ensure a comprehensive assessment of their properties. X-ray Diffraction (XRD) was performed with a measurement range from 2θ of 10° to 50°, with a step size of 0.02° and a time of 5 s per step using the High-Resolution Diffractometer (Zeiss HZGS) at the CBPF (Brazilian Center for Research in Physics). The morphology, size, and nanostructural features of the nanoparticles were examined by transmission electron microscopy (TEM, JEOL-JEM-1011). Fourier transform infrared spectroscopy (FTIR) using KBr pellets was employed to identify functional groups and confirm the presence of carbonate and hydroxyl groups on the nanoparticle. The surface charge and colloidal stability were evaluated by measuring the zeta potential in a 1 mM KCl solution using a Litesizer DLS 500 analyzer. Finally, the hydrodynamic size distribution of the nanoparticles in suspension was determined by dynamic light scattering (DLS) in a NaCl solution at a concentration of 1.2 g/mL.

### 2.5 Cell Culture

Primary murine osteoblasts (f-OST) used in this study were derived from a cell line originally isolated from the endosteal region of adult BALB/c femurs by Balduino et al (2005)^16^ at the Federal University of Rio de Janeiro (UFRJ) and subsequently immortalized. These cells were previously characterized phenotypically and functionally as osteoblastic cells with a tendency toward terminal differentiation. The cells were maintained in DMEM (high glucose) supplemented with 10% FBS and 1% PS and were passaged until they reached sufficient confluence for experimentation.

### 2.6 Optical Tweezers Adhesion Assay

Cells (15,000 cells/cm^2^) were seeded onto glass-bottom dishes (SPL Life Sciences, Korea) and incubated overnight to promote adhesion. Nanostructured CarboHA from each synthesis condition was then added to the culture dish and analyzed using optical tweezers. Adhesion rates were quantified according to previously published methods.^17,18^ Briefly, a CarboHA particle in suspension was captured by the optical tweezers laser and placed in contact with the cell surface for 2, 30, 60, 120, or 240 s. The microscope stage was then displaced to assess particle detachment. Successful detachment was recorded as a negative adhesion event, whereas attachment was considered a positive adhesion event. Relative adhesion was defined as the ratio of positive adhesion events (N/N_0_) at each contact time (t). The characteristic adhesion time (τ), required for approximately 63% of the adhesion events to be positive, was determined by fitting the adhesion curves to the equation: *N*/*N*_0_ = 1–*e* ^(−*t*/*τ*)^. Error bars represent half the range between maximum and minimum values across 30 events, with each event performed in three independent experiments. All data fitting and plots were generated using Kaleidagraph software (Synergy Software, Essex Junction, VT, USA).

### 2.7 Inhibitor Dose Selection

Cells (15,000 cells/cm^2^) were exposed to varying concentrations of endocytosis pathway inhibitors based on literature recommendations. The selected concentrations included: chlorpromazine (100 μM, clathrin-mediated), genistein (300 μM, caveolin-mediated), methyl-β-cyclodextrin (MβCD) (10 mM, lipid rafts), amiloride (100 μM, macropinocytosis), and lovastatin (100 μM, phagocytosis). Pathway inhibition was validated using fluorescent tracers: transferrin (15 μg/mL) for the clathrin-mediated pathway and cholera toxin (5 μg/mL) for the caveolin/lipid raft pathways.

### 2.8 Internalization Assays

Cells (30,000 cells per well) were seeded on glass coverslips in 24-well plates. After the inhibitor treatment, cells were exposed to Rhodamine-labeled CarboHA in DMEM with 10% FBS for either 20 min or 2 h, following protocols adapted from Rossi et al. (2017).

### 2.9 Immunofluorescence Staining

After exposure, cells were fixed with 4% paraformaldehyde, quenched with 20 mM NH_4_Cl, permeabilized with 0.1% Triton X-100, and stained with Alexa Fluor 647 (1:50 in 1% BSA + 0.1% Triton X-100). Nuclei were stained with DAPI. Images were captured using fluorescence microscopy, analyzing 10 fields per sample in triplicate. Quantification of internalized CarboHA was performed via colocalization analysis.

### 2.10 Transmission Electron Microscopy (TEM)

Transmission Electron Microscopy (TEM) was used to evaluate the morphology, structural organization, size, and nanostructural features of the CarboHA samples. For the analysis, we prepared 300-mesh copper grids coated with Formvar, onto which we deposited 10 μL of the sample and allowed it to settle for 10 min. Afterward, we removed the excess liquid. Imaging was conducted using a JEOL-JEM-1011 TEM at the multiuser electron microscopy platform of FIOCRUZ. Individual CarboHA nanoparticles from each synthesis condition were manually selected and analyzed using the Fit Ellipse tool in ImageJ software to determine the elliptical aspect ratio (AR), calculated as the ratio of the major axis to the minor axis. AR values for each condition were then plotted as scatter plots in GraphPad Prism, and the results are presented as mean ± SEM.

### 2.11 Statistical Analysis

For the analysis of internalization and endocytic pathways, we processed images using Leica LAS-X and ImageJ software. Experiments were conducted in triplicate, with 30,000 cells seeded per well on glass coverslips. Ten images from distinct fields were captured per well, resulting in a total of 30 images per experiment. In Leica LAS-X, the colocalization function was used to quantify only the fluorescent CarboHA particles that were internalized within cells, as confirmed by their overlap with phalloidin 647 staining of the cytoskeleton. Subsequently, we used ImageJ to normalize the data by calculating the area occupied by cells in each field, which allowed for the determination of the mean area of CarboHA per cell.

Statistical analyses were performed using GraphPad Prism software (version 10). Initially, data normality was assessed using the Shapiro–Wilk test. Comparisons between groups were carried out using Student’s *t* test or ANOVA, with post hoc tests applied when necessary. p-values are indicated in the legends of each figure.

## 3 Results

### 3.1 Characterization of Carbo-Hydroxyapatite Samples

Nanostructured carbonated hydroxyapatite (CarboHA) was synthesized by wet chemical precipitation at three distinct temperatures: 5 °C, 37 °C, and 90 °C. The resulting powders exhibited noticeable differences in agglomeration behavior depending on the synthesis temperature. CarboHA obtained at 5 °C presented a fine, homogeneously dispersed powder, whereas samples synthesized at higher temperatures (37 and 90 °C) formed progressively larger agglomerates, consistent with the intrinsic tendency of this biomaterial to cluster during precipitation.

Representative optical and scanning electron microscopy images illustrating the macroscopic morphology and agglomeration behavior of CarboHA synthesized at different temperatures are provided in the Supporting Information (Figure S1). These images corroborate the observed temperature-dependent differences in powder dispersion and agglomerate formation described above.

The crystallographic structure and phase composition were analyzed by X-ray diffraction (XRD), and the diffraction patterns are presented in Figure 1. These diffractograms exhibit only peaks of crystalline phase referent to CIF (26024-ICSD). The samples exhibited characteristic peaks of hydroxyapatite, corresponding to the (002), (112), (210), (211), (300), (310), (222), and (213) planes, in agreement with reference data from the literature,^19^ confirming the successful synthesis of the material at all temperatures. The sample prepared at 5 °C displayed broader and less intense peaks, indicative of reduced crystallinity and smaller crystallite size, while those synthesized at 37 and 90 °C showed increasingly sharper reflections, particularly at higher temperatures, suggesting enhanced long-range order and crystal domain growth.

**Figure 1:**
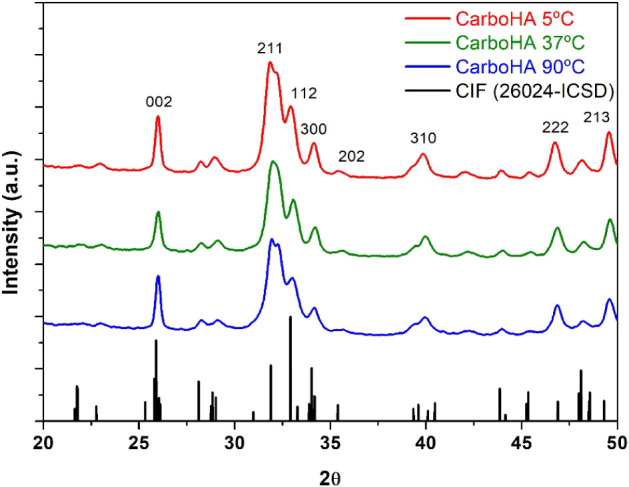
XRD graph generated by the analysis of carbonated hydroxyapatite, confirming the biomaterial as HA. The similarity between the samples can be observed.

The variation in the nanoparticle size as a function of synthesis temperature was confirmed by TEM. Higher synthesis temperatures resulted in a clear increase in the nanoparticle size (Figure 2). In addition, the elliptical aspect ratio (AR) of CarboHA nanoparticles was measured and showed a significant increase with synthesis temperature (Figure 3).

**Figure 2:**
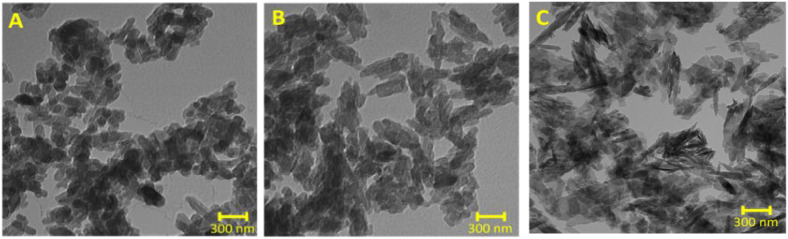
Nanostructured CarboHA viewed by TEM. (A) CarboHA synthesized at 5 °C; (B) CarboHA synthesized at 37 °C; (C) CarboHA synthesized at 90 °C.

**Figure 3:**
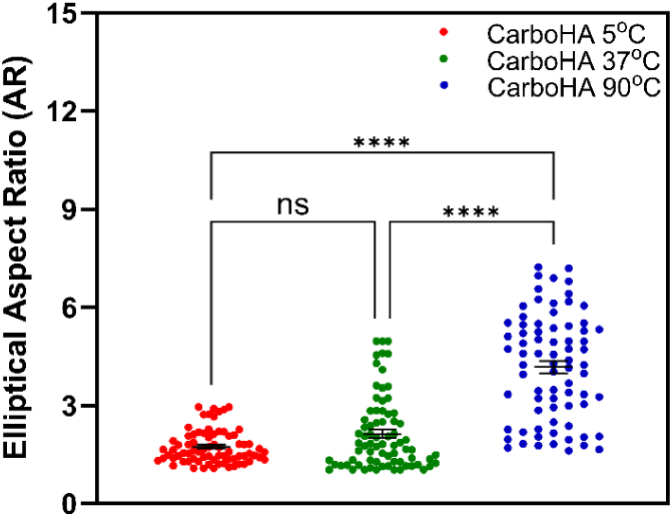
Quantitative plot showing the elliptical aspect ratio parameter as a function of the synthesis temperature. Each point represents a measurement. The black horizontal bars indicate the means, while vertical bars represent the standard error of the mean (SEM). Statistical analysis was performed using one-way ANOVA followed by Tukey’s posttest. ns means *p* > 0.05; **** means *p* < 0.0001.

### 3.2 Fluorescent Rhodamine Labeling of Carbonated Hydroxyapatite Nanoparticles

Fluorescent CarboHA nanoparticles were obtained by Rhodamine adsorption, a fluorophore with characteristic emission at 575 nm. The successful conjugation was visually confirmed by the color change from white to pink, while preserving the morphological differences related to synthesis temperature (Figure 4). The fluorescent labeling of CarboHA with Rhodamine was confirmed by fluorescence measurements, which demonstrated successful adsorption of the fluorophore onto the nanomaterials. Quantitative estimates of the fluorescent loading for CarboHA synthesized at different temperatures are provided in the Supporting Information in Figure S2.

**Figure 4:**
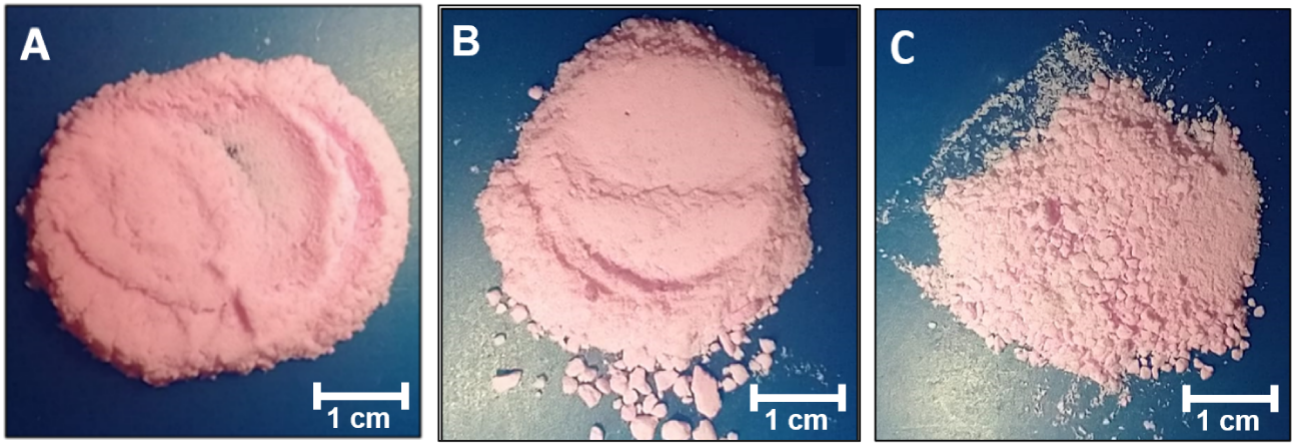
Visual aspect of Rhodamine adsorbed in nanostructured carbonated hydroxyapatite. (A) Synthesized at 5 °C; (B) Synthesized at 37 °C; (C) Synthesized at 90 °C.

Fourier Transform Infrared Spectroscopy (FTIR) was performed to identify the functional groups after Rhodamine adsorption, confirming both the adsorption and the preservation of the nanoparticle’s chemical structure. Characteristic hydroxyl and phosphate groups of hydroxyapatites were identified, along with carbonate groups (CO_3_^2–^) indicated by absorption bands at 1400–1600 cm^–1^, confirming B-type carbonate substitution, and verifying successful carbonation without alteration of the biomaterial post-Rhodamine adsorption. These findings align with reference spectra from Lara-Ochoa et al.^19^ The presence of Rhodamine was confirmed by a band around 2900–3000 cm^–1^, consistent with literature.^20,21^ These results confirm successful Rhodamine adsorption without compromising the nanoparticle’s chemical integrity (Figure 5).

**Figure 5:**
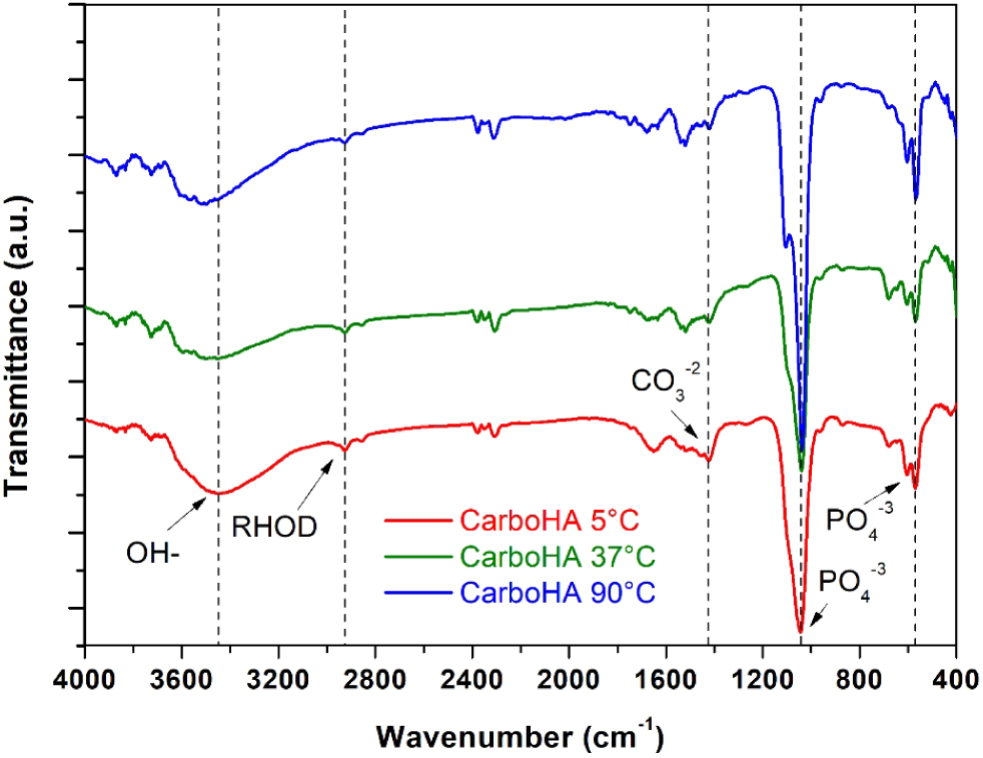
Resulting FTIR graphs, confirming the carbonation and the unchanged nature of CarboHA by Rhodamine.

Zeta potential analysis was performed to evaluate surface charge changes related to protein adsorption before and after Rhodamine conjugation and following exposure to DMEM supplemented with 10% FBS (Figure 6). Exposure to the culture medium significantly increased protein adsorption due to the presence of serum proteins. In water, CarboHA samples without Rhodamine exhibited zeta potentials of −11.4 mV (5 °C), −12.3 mV (37 °C), and −13.2 mV (90 °C). After Rhodamine adsorption, these values decreased to −19.5 mV, −19.9 mV, and −17.6 mV, respectively, reflecting the modification of the nanoparticle surface. Upon incubation with DMEM + 10% FBS, a marked increase in negative surface charge was observed, with zeta potentials of −28.1 mV (5 °C), −29.7 mV (37 °C), and −28.1 mV (90 °C) for unlabeled samples. Rhodamine-labeled nanoparticles showed further increases to −35.6 mV, −37.2 mV, and −37.3 mV, indicating enhanced protein adsorption likely due to both serum components and the additional surface features introduced by Rhodamine.

**Figure 6:**
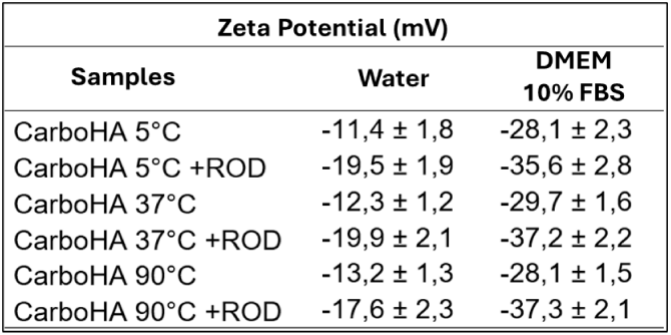
Zeta potential analysis, comparing the samples synthesized at the same temperature regarding protein adsorption before and after exposure to the culture medium with FBS, including pure samples and those already adsorbed with Rhodamine. There was a significant increase in potential in the samples exposed to the culture medium, and in both cases, Rhodamine caused a slight increase in potential.

### 3.3 Temporal Dynamics and Uptake Mechanisms of Carbonated Hydroxyapatite

Optical tweezers-based adhesion assays revealed that the characteristic adhesion time (τ) of CarboHA nanoparticles increased with synthesis temperature, rising from 59.6 ± 4.5 s at 5 °C, to 67.1 ± 4.5 s at 37 °C, and reaching 98.6 ± 10.4 s at 90 °C (Figure 7).

**Figure 7:**
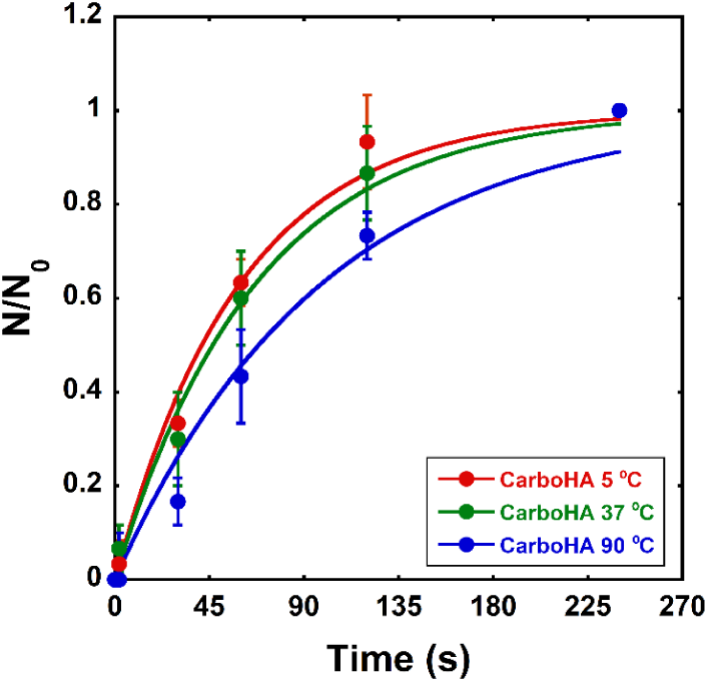
Optical tweezers adhesion assays showed increasing adhesion times with synthesis temperature: 59.6 s (5 °C), 67.1 s (37 °C), and 98.6 s (90 °C).

The internalization assays demonstrated that the cellular uptake of carbonated hydroxyapatite (CarboHA) is markedly influenced by the synthesis temperature, with each condition showing a distinct endocytic profile. For CarboHA synthesized at 5 °C, internalization was heavily dependent on clathrin- and caveolin-mediated endocytosis. Inhibition of these pathways led to drastic reductions in uptake, dropping to 16% and 35% of the control, respectively. By contrast, inhibition of macropinocytosis and phagocytosis only modestly reduced internalization, which remained at 83% and 87%, indicating that these two routes play only minor roles for CarboHA at this synthesis temperature.

CarboHA produced at 90 °C displayed the opposite trend. Internalization was largely unaffected by inhibition of clathrin- or caveolin-mediated pathways, with uptake maintained at nearly 90% of the control. However, macropinocytosis and phagocytosis proved critical for these particles, as inhibition of either pathway drastically reduced internalization to 35% and 27%, respectively. These findings suggest that larger and more crystalline particles rely predominantly on these two endocytic routes to enter cells.

An intermediate behavior was observed for CarboHA synthesized at 37 °C. Inhibition of any of the four pathways resulted in a moderate reduction in internalization, with uptake levels ranging between 50% and 60% of the control. This pattern indicates that CarboHA at physiological synthesis temperature does not rely on a single endocytic route, but rather uses a combination of clathrin-, caveolin-, macropinocytosis-, and phagocytosis-mediated pathways to penetrate cells.

Finally, lipid raft-mediated endocytosis did not significantly contribute to the uptake of CarboHA, regardless of synthesis temperature. Inhibition of this pathway produced no measurable changes, with internalization remaining between 90% and 100% across all conditions. These results demonstrate a clear relationship between synthesis temperature, nanoparticle characteristics, and the endocytic mechanisms involved in cellular uptake (Figure 8A). Representative fluorescence microscopy images of F-OST cells further illustrate these differences (Figure 8B).

**Figure 8:**
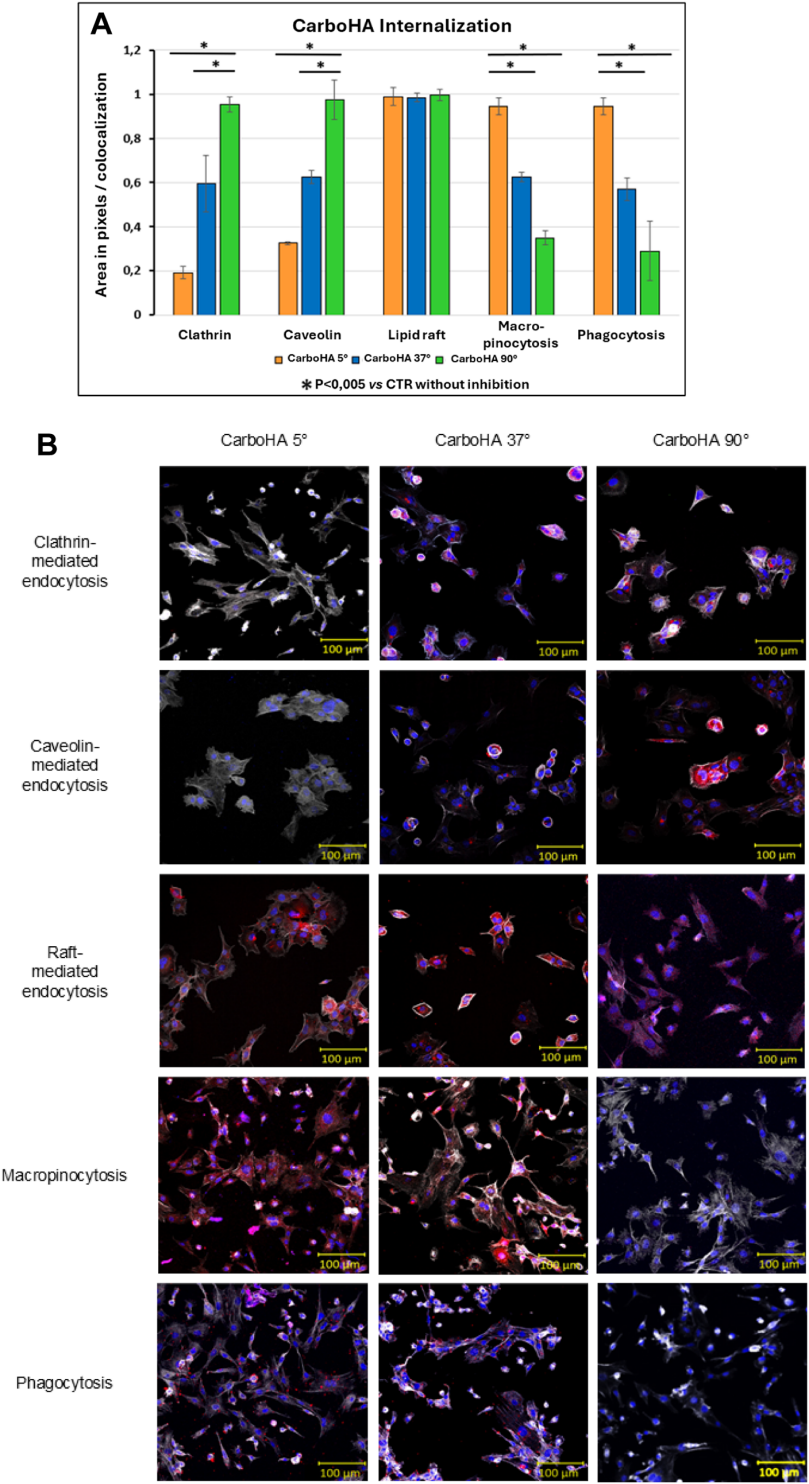
(A) Comparative graphs of the different endocytic pathways. CarboHA 5 °C has its internalization inhibited when the clathrin and caveolin pathways are blocked; CarboHA 37 °C has only half of its internalization affected; CarboHA 90 °C had its internalization rate decreased in the macropinocytosis and phagocytosis pathways. Among the five pathways, the only one that did not show any alteration was the lipid raft pathway, indicating that it is not used for CarboHA internalization. (B) Representative fluorescence microscopy images of F-OST cells exposed to carbonated hydroxyapatite nanoparticles following inhibition of different endocytic pathways.

## 4 Discussion

Calcium hydroxyapatite-based materials are well established in clinical practice across several biomedical domains. In aesthetic medicine, CarboHA fillers such as Radiesse serve as biostimulators, providing immediate volume and promoting longer-term collagen remodeling to enhance skin texture and facial contouring; recently published studies sustained volumetric improvement and enhanced skin hydration and elasticity following CarboHA injections.^22^ In dentistry, nanohydroxyapatite formulations are increasingly used for enamel remineralization, dentin desensitization, and as active ingredients in preventive oral care products. Clinical evidence underscores hydroxyapatite’s efficacy in caries prevention and dentin hypersensitivity control.^23^

In bone regeneration, carbonate-containing apatites and other HA-based bioceramics function as osteoconductive graft materials and as constituents of composite bone substitute systems. Reviews and investigations highlight the superior osteoconductivity and clinical promise of CarboHA for ridge augmentation and critical-size defect repair.^24,25^

This study offers new insights into the adhesion/internalization mechanisms of nanostructured carbonated hydroxyapatite (CarboHA), which was synthesized through wet precipitation at low temperatures. While previous research has thoroughly characterized hydroxyapatite produced using high-temperature, dry methods, the behavior of CarboHA prepared under physiological conditions, specifically at 5 and 37 °C, remains poorly understood.

The particle size and nanostructural characteristics of CarboHA synthesized using the protocol adapted from Anjos et al. (2019) was previously characterized in detail in the same study, in which high-resolution transmission electron microscopy (TEM) was used to evaluate particle morphology and size, and quantitative measurements were obtained by analyzing individual crystals with ImageJ software based on measurements of 30 particles per sample, providing baseline structural data for comparison with the current work.

Because this biomaterial consists of nanostructured agglomerates with a broad particle size distribution, the determination of fluorescent loading capacity is inherently imprecise and the fluorescence intensity is strongly influenced not only by the number of fluorophores present, but also by particle aggregation, quenching/dequenching effects, and local signal saturation, all of which can vary with particle size and environment. Recent studies have highlighted that fluorescence signals can be unreliable for absolute quantification of nanoparticle loadings, particularly when particle size, dye distribution, or quenching phenomena are not uniform across samples, leading to potential under- or overestimation of cargo content.^26^

Our findings suggest that synthesis temperature influences not only CarboHa’s physicochemical properties (e.g., crystallinity, particle size and morphological aspects) but also its cellular adhesion/internalization features. These effects are likely mediated by changes in surface energy and particle morphology, both of which impact the interaction between nanoparticles and cell membranes.

Lower synthesis temperatures resulted in materials with reduced crystallinity, which is associated with increased solubility and reactivity.^27,28^ Wet precipitation refers to synthesis carried out at temperatures below the evaporation point of water, allowing mineral precipitation to occur directly in an aqueous medium under controlled thermal conditions. In the present study, low-temperature wet precipitation corresponds to 5 °C, whereas medium- and high-temperature syntheses commonly reported in the literature correspond to 37 and 90 °C, respectively.

Low-temperature wet precipitation limits crystal growth and favors the formation of nanostructured carbonated hydroxyapatite with higher surface area, lower crystallinity, and enhanced chemical reactivity, features that are particularly relevant for studies focused on nanoparticle–cell interactions. Previous studies comparing synthesis temperatures have demonstrated that hydroxyapatite produced under lower thermal conditions exhibits smaller crystal size and reduced crystallinity relative to materials synthesized at elevated temperatures, directly impacting surface properties and biological behavior.^29^ In contrast, conventional wet precipitation conducted at higher temperatures tends to increase crystal size and crystallinity, which can reduce surface area and adversely affect biological interactions, including cell viability and adhesion.^30^

The reduced crystallinity associated with low-temperature synthesis may enhance biological performance by promoting ion release and facilitating interactions with intracellular compartments. Moreover, low-crystalline hydroxyapatite has been linked to improved protein adsorption and superior bone integration, reinforcing its suitability for applications requiring active biological engagement.^31,32^ These characteristics are particularly important for enhancing cellular adhesion, proliferation, and ultimately facilitating bone regeneration.^14^ Although Zeta potential analyses in this study did not reveal significant differences in surface charge, this may reflect the method’s limitations in quantifying protein corona composition, highlighting the need for further proteomic investigations.

Endocytic profiling showed that CarboHA internalization is not uniform across different synthesis temperatures. The less crystalline nanoparticles (e.g., CarboHA synthesized at 5 °C) were mainly internalized through clathrin- and caveolin-mediated pathways, which align with the vesicle size range typically associated with these mechanisms.^33^ Conversely, CarboHA synthesized at 90 °C exhibited predominant uptake through macropinocytosis and phagocytosis. These findings are consistent with prior research demonstrating that particle size is a significant factor in determining endocytic routing.^34^ Interestingly, the adhesion assays mirrored the internalization trends and correlated with quantitative morphological features. Optical tweezers measurements revealed that the characteristic adhesion time increased progressively with synthesis temperature, from about 60 s for CarboHA prepared at 5 °C to nearly 100 s for the sample synthesized at 90 °C. Such increase likely reflects the higher surface crystallinity and reduced curvature of larger particles. Conversely, smaller and less crystalline nanoparticles exhibited shorter adhesion times. CarboHA produced at 37 °C demonstrated partial internalization across multiple pathways, suggesting an intermediate behavior that may represent a favorable balance between stability and bioactivity. This observation aligns with in vivo studies indicating improved osteointegration for materials synthesized under similar conditions.^35^

Evidence indicating that cellular adhesion is not merely a preliminary event prior to endocytosis, but an active contributor to the selection and efficiency of internalization mechanisms is plentiful. Adhesion between nanoparticles and the cell membrane involves a combination of physicochemical factors, including surface energy, particle curvature, and receptor–ligand interactions, which together determine the degree and duration of membrane contact prior to uptake.^36^ Recent studies have highlighted that stronger or longer adhesion increases the likelihood of membrane wrapping and recruitment of endocytic machinery, such as the clathrin and caveolin systems, while weaker adhesion may favor alternative uptake mechanisms or incomplete wrapping.

For example, comparative analyses across nanomaterials reveal that particle size and surface features directly influence how cells wrap and internalize nanoparticles. Larger or more rigid nanoparticles tend to sustain prolonged membrane contact, which promotes actin-mediated processes such as macropinocytosis and phagocytosis, consistent with our observation of predominant uptake of higher-crystallinity, larger CarboHA at 90 °C through these pathways.^37^ Conversely, smaller particles with higher surface energy and curvature enable more efficient initiation of receptor-mediated pathways like clathrin- and caveolin-mediated endocytosis, aligning with our findings for CarboHA synthesized at 5 °C. A study on iron oxide nanorods systematically demonstrated that variations in nanoparticle size and morphology altered the relative contributions of clathrin, caveolae, and phagocytic mechanisms, underscoring the interplay between adhesion energetics and internalization route preferences.^38^

Carbonate substitution and low crystallinity further modulate these interactions by increasing surface reactivity and lowering rigidity, which may enhance initial adhesion through increased protein adsorption and dynamic protein corona effects. Recent reviews emphasize that protein corona composition and surface chemistry influence both adhesion strength and endocytic responses, with more reactive surfaces facilitating wetter membrane contact and receptor engagement.^36^ Therefore, the synthesis-dependent variation in surface characteristics we observe provides a mechanistic basis for the correlated patterns of adhesion time and pathway preference: longer adhesion events bias toward uptake routes requiring extensive membrane remodeling (e.g., macropinocytosis), whereas shorter but firm adhesion favors clathrin/caveolin recruitment and vesicle formation.

Importantly, these distinctions are not merely academic; understanding the mechanisms by which nanoparticles enter cells is crucial for optimizing their design for clinical applications, particularly in drug delivery and bone regeneration. This research also helps fill a gap in the literature by systematically correlating low-temperature synthesis of CarboHA with its cellular uptake behavior. Since bone mineralization *in vivo* occurs under aqueous, moderate-temperature conditions,^39^ our synthesis approach may better mimic physiological mineral phases compared to traditional high-temperature methods.

This work’s demonstration of synthesis-dependent control over particle size and crystallinity enables the tuning of dominant cellular uptake pathways. Because the endocytic route adopted by the cell influences the intracellular fate of the material, its presentation to osteoblasts, and the potential for therapeutic payload delivery, this tunability opens clear avenues for engineered nanocarriers and bioactive grafts. It was shown that nanoparticle size, crystallinity and surface chemistry strongly determine protein corona formation and endocytic routing, factors intimately linked to bioactivity and safety.^22–31^ By using wet, low-temperature processing closer to physiological mineralization, CarboHA may yield a mineral phase that more closely mimics bone apatite, thereby enhancing cell recognition, matrix integration, and regulatory alignment for medical devices emphasizing biomimicry.^25,40^

In practical terms, especially for bone grafts and substitute materials, these features create compelling opportunities. CarboHA could serve as a resorbable graft material tailored for clinical situations requiring staged remodeling.

By adjusting synthesis conditions to produce smaller, less crystalline particles that promote clathrin- and caveolin-mediated uptake and faster intracellular processing, or larger, more crystalline particles that rely on macropinocytosis and phagocytosis for slower degradation, it becomes possible to design grafts precisely aligned with the defect’s natural healing timeline.^31–41^ Moreover, the enhanced solubility and cellular uptake suggest that CarboHA may outperform conventional HA grafts by accelerating early bone formation and remodeling while maintaining osteoconductivity, a notion supported by a preclinical findings where low-crystalline CarboHA facilitated improved bone ingrowth and macrophage-mediated osteogenic environments.^42^

Thus, the mechanistic link established in this study between synthesis temperature, nanostructure, and uptake pathway provides actionable design rules for developing application-specific CarboHA products, ranging from resorbable bone grafts for defect repair to nanocarriers for bioactive agent delivery, offering superior performance due to their synthesis under near-physiological conditions and optimization for controlled cellular processing.

## 5 Conclusions

This study examined the internalization and intracellular trafficking of carbonated hydroxyapatite (CarboHA) nanoparticles synthesized through the wet route at three different temperatures: 5 °C, 37 °C, and 90 °C, in primary murine osteoblast cultures. The synthesis was successful at all tested temperatures, yielding materials with distinct physicochemical characteristics influenced by the synthesis temperature. Specifically, both crystallinity and primary nanoparticle size increased with temperature. Fluorescent labeling was achieved through the adsorption of Rhodamine, allowing for tracking without altering the fundamental nature of the biomaterial. Zeta potential analyses demonstrated the formation of a protein corona when exposed to biological media, regardless of the synthesis temperature. Importantly, adhesion/internalization pathways were shown to vary according to particle characteristics: CarboHA synthesized at 5 °C showed the faster adhesion time and was internalized primarily via clathrin- and caveolin-mediated endocytosis; CarboHA at 90 °C exhibited the slower adhesion time and favored micropinocytosis and phagocytosis; and the intermediate sample, synthesized at 37 °C, presented the intermediate adhesion time and employed all major pathways. Notably, lipid raft-mediated endocytosis did not appear to be a significant route for any of the CarboHA samples tested. These findings emphasize the potential to tailor nanoparticle-cell interactions through controlled synthesis parameters, providing valuable insights for future biomedical applications of CarboHA.

## Supplementary Material



## Notes

Particle size and hydrodynamic distribution of carbonated hydroxyapatite nanoparticles determined by dynamic light scattering (DLS); dispersion protocol by ultrasonication prior to cell exposure; fluorescence characterization of rhodamine-labeled CarboHA; calibration curve and emission profiles for determination of rhodamine loading in CarboHA synthesized at different temperatures (PDF)
