# Supplementary material for: Temporal Dynamics and Uptake Mechanisms of Carbonated Hydroxyapatite Nanoparticles in Murine F‑OST Cells
Source: ACS Omega. 2026 Mar 18;11(12):19282–91. doi: 10.1021/acsomega.5c12494 (PMC13044684; doi:10.1021/acsomega.5c12494)
Supplement: Supplementary file 1 [file ao5c12494_si_001.pdf]

TEMPORAL DYNAMICS AND UPTAKE MECHANISMS OF CARBONATED HYDROXYAPATITE NANOPARTICLES IN MURINE F-OST CELLS

Julliana Helena de Souza Sobrinho[a], Marcel Guimarães Martins[b], Grasiella Ventura Matioszek[c], Alexandre Malta Rossi[d], Bruno de Almeida Carlos de Carvalho Pontes[e], Fabrício Frizera Borghi[f], Danielle Cabral Bonfim[g], Sara Gemini-Piperni*

[a] *University of Grande Rio, Duque de Caxias 25075-142, Rio de Janeiro, Brazil.*

[b] *MAGTECH BRASIL, business incubator of Federal Fluminense University, 24220-000, Rio de Janeiro, Brazil.*

[c] *Institute of Biomedical Sciences – ICB, Federal University of Rio de Janeiro, Rio de Janeiro 21941-590, Brazil.*

[d] Brazilian Center of Physical Research – *CBPF, Botafogo, Rio de Janeiro - RJ, 22290-180, Brazil.*

[e] *Institute of Biomedical Sciences – ICB and National Center for Structural Biology and Bioimaging – CENABIO, Federal University of Rio de Janeiro, Rio de Janeiro 21941-590, Brazil.*

[f] *Institute of Physics – ICB, Federal University of Rio de Janeiro, Rio de Janeiro 21941-590, Brazil.*

[g] *Institute of Biomedical Sciences – ICB, Federal University of Rio de Janeiro, Rio de Janeiro 21941-590, Brazil.*

* *Institute of Biomedical Sciences* – ICB, Federal University of Rio de Janeiro, Rio de Janeiro 21941-590, Brazi. Email: sara.gemini@icb.ufrj.br

1. PARTICLE SIZE AND HYDRODYNAMIC DISTRIBUTION OF CARBONATED HYDROXYAPATITE NANOPARTICLES

Due to the high agglomeration tendency of carbonated hydroxyapatite, a dispersion protocol using ultrasonication was applied prior to cell exposure. Nanoparticles were sonicated for 15 minutes at 99% of 750 W power at a concentration of 1 mg/mL. Dynamic Light Scattering (DLS) analysis confirmed a substantial reduction in aggregate size, with an average hydrodynamic diameter ranging between 2–3 µm (Figure 1).

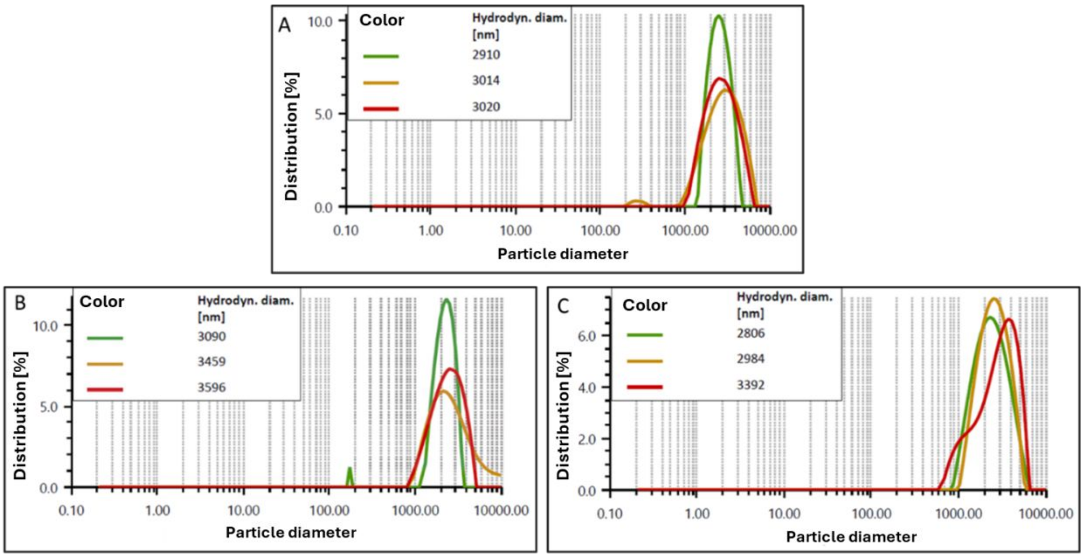

Figure S1: (A) Carbonated hydroxyapatite synthesized at 5°C with an average hydrodynamic diameter of 2.5µ (2500nm); (B) Carbonated hydroxyapatite synthesized at 37°C with an average hydrodynamic diameter of 2.5µ (2500nm); (C) Carbonated hydroxyapatite synthesized at 90°C with an average hydrodynamic diameter of 2µ (2000nm).

2. FLUORESCENCE CHARACTERIZATION OF RHODAMINE-LABELED CARBOHA

Fluorescence measurements of CarboHA with adsorbed Rhodamine (resuspended nanomaterials in water) allowed for the estimation of the fluorophore load adsorbed onto the nanomaterial: 33.3 µg/g to CarboHA 5 ºC, 113.3 µg/g to CarboHA 37 ºC and 66.7 µg/g to CarboHA 90 ºC. The fluorescent loading capacity for the Carbo HA confirms the fluorescent labeling of the nanomaterials, ideal for cell uptake assays. It is worth noting that the difference in fluorescence intensity between Rhodamine-labeled CarboHA does not influence subsequent cell uptake assays, since internalization will be determined by the area of the cell occupied by the fluorescent nanomaterial.

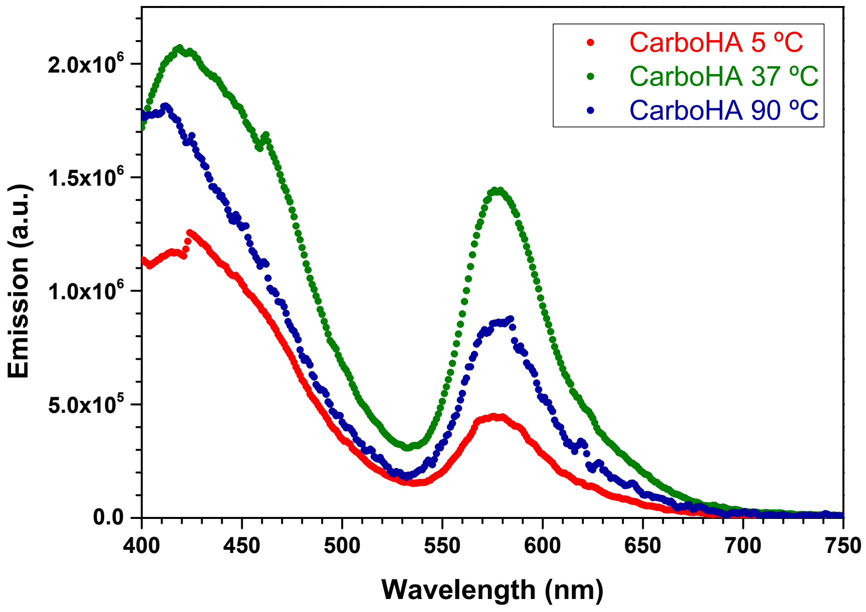

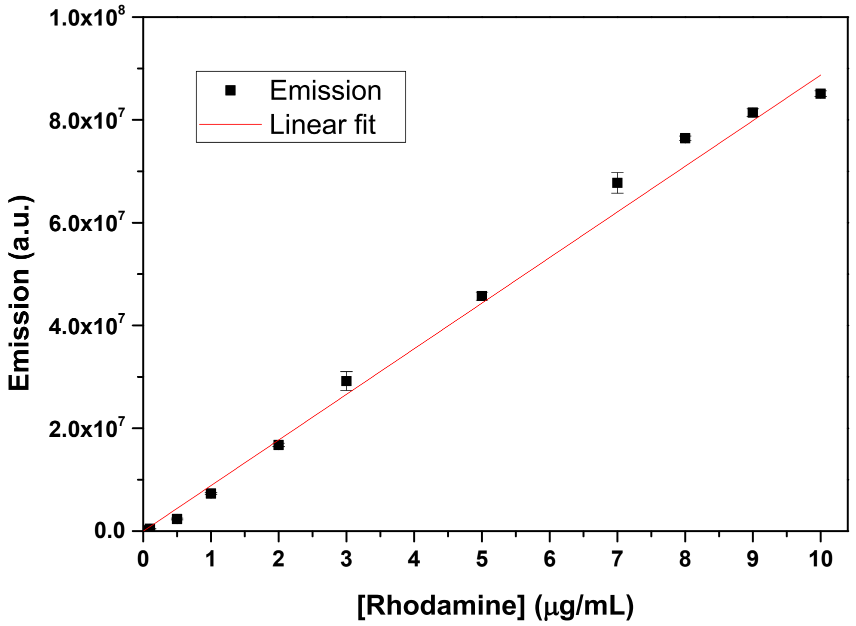

Figure S2: Emission profile of CarboHA with Rhodamine adsorbed (suspension at 1.5 mg/mL), confirming the fluorescence of the nanomaterials. The calibration curve allows determining the rhodamine load in CarboHA: 33.3 μg/g to CarboHA 5 ºC, 113.3 μg/g to CarboHA 37 ºC and 66.7 μg/g to CarboHA 90 ºC.
